# Novel multifunctional pH-sensitive nanoparticles loaded into microbubbles as drug delivery vehicles for enhanced tumor targeting

**DOI:** 10.1038/srep29321

**Published:** 2016-07-05

**Authors:** Yongjiu Lv, Lan Hao, Wenjing Hu, Ya Ran, Yan Bai, Liangke Zhang

**Affiliations:** 1 Chongqing Research Center for Pharmaceutical Engineering, School of Pharmacy, Chongqing Medical University, Chongqing 400016, P.R. China; 2 Chongqing Key Laboratory of Ultrasound Molecular Imaging, Institute of Ultrasound Imaging, Chongqing Medical University, Chongqing 400016, P.R. China; 3 Chongqingshi Shapingba District People’s Hospital, Chongqing 400030, P.R. China

## Abstract

This study fabricated novel multifunctional pH-sensitive nanoparticles loaded into microbubbles (PNP-MB) with the combined advantages of two excellent drug delivery vehicles, namely, pH-sensitive nanoparticles and microbubbles. As an antitumor drug, resveratrol (RES) was loaded into acetylated β-cyclodextrin nanoparticles (RES-PNP). The drug-loaded nanoparticles were then encapsulated into the internal space of the microbubbles. The characterization and morphology of this vehicle were investigated through dynamic light scattering and confocal laser scanning microscopy, respectively. *In vitro* drug release was performed to investigate the pH sensitivity of RES-PNP. The antitumor property of RES-loaded PNP-MB (RES-PNP-MB) was also analyzed *in vivo* to evaluate the antitumor effect of RES-PNP-MB. Results suggested that PNP exhibited pH sensitivity, and was successfully encapsulated into the microbubbles. RES-PNP-MB exhibit effective tumor growth suppressing *in vivo*. Therefore, such drug delivery vehicle should be of great attention in tumor therapy.

Cancer is a life-threatening disease with high morbidity and mortality^1^,^2^,^3^. This disease is conventionally treated through chemotherapy. However, most anticancer drugs exhibit high-dose-limiting toxicities and narrow therapeutic windows because of the poor specificity of drugs and inadequate targeting ability of treatments^4^,^5^,^6^. Compared with conventional medicines, nanoparticle drug delivery systems provide several advantages, including passive/active targeting, controlled or sustained drug-release kinetics, and improved patient compliance; these systems can also enhance the physicochemical properties of drugs because of their improved permeability and retention (EPR) effect, modifiable external shell, specific nanomaterial properties, and low cytotoxicity^7^,^8^,^9^,^10^,^11^,^12^,^13^,^14^,^15^,^16^.

Among nanoparticles, pH-sensitive ones have been widely used in cancer therapy. The tumor extracellular space in poorly perfused regions is highly acidic compared with the surrounding normal tissues because of the high metabolic rate and inadequate oxygen supply of tumor cells^17^. pH-sensitive nanoparticles, which are designed to be activated by low pH, release the loaded drugs into the acidic extracellular space of solid tumors; in this process, the encapsulated drugs are stabilized during blood circulation until nanoparticles passively accumulate in tumor sites via the EPR effect^18^,^19^,^20^,^21^,^22^,^23^,^24^,^25^,^26^.

Gas-filled microbubbles have been used as ultrasound contrast agents to improve the quality of images and to extend the diagnostic scope of ultrasound imaging^27^,^28^. Microbubbles can be destroyed through focused ultrasound irradiation; these microbubbles have been investigated as potential gene or drug vehicles for organ- and tissue-specific drug delivery^29^,^30^. They can improve the drug efficacy and reduce the undesired side effects by enhancing vessel permeability and ultrasound-triggered drug release in target regions because of their bioeffect^31^,^32^,^33^,^34^.

In this study, pH-sensitive nanoparticle-loaded microbubble (RES-PNP-MB; Fig. 1), a novel multifunctional drug delivery vehicle, was prepared to maximize the targeting effect and to minimize the undesired side effects of antitumor drugs. As an antitumor drug, resveratrol (RES) can induce the S phase arrest of H22 cells^35^; this drug was loaded into the pH-sensitive nanoparticles. The RES-loaded pH-sensitive nanoparticles (RES-PNP) were designed to be encapsulated in the microbubbles and designated as RES-PNP-MB. This design can enhance the targeting effect and reduce the target-off and systemic side effects (Fig. 1).

The microbubbles were used to transport the loaded RES-PNP to the target tumor site; this procedure could be visually monitored through ultrasound imaging. This approach could protect RES-PNP from drug leakage and could prevent the degradation of the pH-sensitive nanoparticles during blood circulation. The pH-sensitive nanoparticles were also introduced to stimulate the site-specific drug release triggered by the low pH condition of the tumor interstitium. The characteristics, *in vitro* drug release, *in vivo* antitumor activity of RES-PNP-MB were further investigated.

## Results and Discussion

### Material synthesis and characterization of PNP, microbubbles, and PNP-MB

A-CD was synthesized via an acetonation reaction between the linear acetal of β-CD and the cyclic acetal of 2-methoxypropene in accordance with a previously described method^36^. PNP was prepared through emulsion/solvent evaporation, and PNP-MB was prepared by mechanical shaking^37^.

Figure 2A illustrates the typical digital photos of PNP, blank microbubbles, physical mixture of PNP and blank microbubbles, and PNP-MB after 3 h of standing. PNP appeared uniformly turbid. The physical mixture was cloudy, while its upper layer was more turbid. The blank microbubbles and PNP-MB moved to the upper layers. By contrast, the lower layers were clearer than the turbid bottom layer of the physical mixture possibly because the blank microbubbles and PNP-MB accommodated fluorocarbon gas that reduced their density. This phenomenon also suggested that PNP was mostly encapsulated in the microbubbles.

We obtained 91.4 ± 6.0 nm PNP, 1924.3 ± 55.8 nm microbubbles, and 2435.7 ± 59.2 nm PNP-MB at pH 7.4. The diameter of PNP-MB was larger than that of microbubbles (Fig. 2B–D). This finding implied that PNP was successfully encapsulated into the microbubbles. The EEs of RES-PNP and RES-PNP-MB were 95.2% ± 1.2% and 78.14% ± 2.16%, respectively.

Confocal laser scanning microscopy (CLSM) was used to investigate the framework and morphology of PNP-MB. In Fig. 3, the green fluorescence of coumarin-6 labeled PNP (C-6-PNP) was located in the internal space of the microbubbles and broadly distributed in the microbubbles. On the basis of the CLSM results, we further confirmed that PNP was successfully encapsulated into the internal space of the microbubbles.

### *In vitro* hydrolysis and release

The *in vitro* hydrolysis of PNP was investigated in PBS solutions with pH 7.4 and 5.0. In Fig. 4, PNP exhibited remarkable pH-sensitive properties. PNP is stable at pH 7.4 and does not undergo hydrolysis for a long time. By contrast, PNP is rapidly and completely hydrolyzed at pH 5.0 within 2 h (Fig. 4).

The results of *in vitro* RES release from RES-PNP and RES-PNP-MB in PBS solutions with different pH values are shown in Fig. 5. The release of RES from RES-PNP was significantly faster at pH 5.0 than at pH 7.4 (*p* < 0.05). This finding demonstrated the pH-sensitive property of RES-PNP.

RES-PNP-MB exhibited slower release times in both PBS solutions (pH = 5.0, 7.4) than RES-PNP did. This phenomenon may occur because RES-PNP was encapsulated into the microbubbles and protected by their lecithin membrane; as a result, the hydrolysis of PNP was delayed. However, the RES release from RES-PNP-MB was significantly increased after application of ultrasound irradiation, indicating that ultrasound irradiation could promote the diffusion of PNP via the ultrasound destruction of lipid membrane of microbubbles.

### *In vitro* and *in vivo* ultrasound imaging

The ultrasound images of the degasssed saline (control), microbubbles, and RES-PNP-MB were captured for *in vitro* ultrasound imaging. The brighter area indicates the enhancement of echo intensity. Compared with the black background of degassed and deionized water, this area can be distinctly observed in the microbubbles and RES-PNP-MB (Fig. 6). Microbubbles and RES-PNP-MB also brought about an enhanced US imaging in *in vivo* ultrasound imaging experiment. These results suggested that RES-PNP-MB retained the ultrasound imaging capacity of microbubbles; as such, RES-PNP-MB could deliver RES to the target region under the guidance of ultrasound imaging.

### *In vivo* antitumor study

The *in vivo* antitumor effects of RES-PNP-MB were investigated in H22 tumor-bearing mice. Figure 7 illustrates the tumor growth curve of H22 tumor-bearing mice after the treatments were administered. The H22 tumor volume was measured by using a Vernier caliper, and the mice were observed 4 days after inoculation. The tumor rapidly grew in group I. The treatments in groups II to VI effectively delayed the tumor growth compared with group I (control). The tumor inhibition rates are shown in Table 1. Group VI yielded the highest tumor inhibition rate and the smallest tumor volume (Fig. 8).

To further evaluate the effects and biosecurity of the test preparation, the main organs and tumors of all groups (group I-VI) were excised and stained with HE for histopathological analyses. The photos of the HE slices were displayed in Fig. 9. Pathological examinations were used to measure the histological structures of each organ and tumor.

As shown in Fig. 9, histological assessment revealed that the tumors from group VI (RES-PNP-MB + US) exhibited extensive necrosis; by contrast, more viable cells were observed in the five other groups.

The organs of group I (control group) and group II (PNP-MB + US) after HE staining showed the relative normal histological structures. However, all the RES preparation-treated groups (group III-VI) displayed different degrees of pathological changes. Free RES and RES-PNP exhibited a certain extent of damage to all the test organs. However, when RES-PNP were loaded into the microbubbles, the toxicity of the resultant RES-PNP-MB was reduced significantly. RES-PNP-MB with ultrasound treatment showed almost no toxicity to all the test organs, indicating the high biosecurity of RES-PNP-MB with ultrasound treatment.

**Figure 1:**
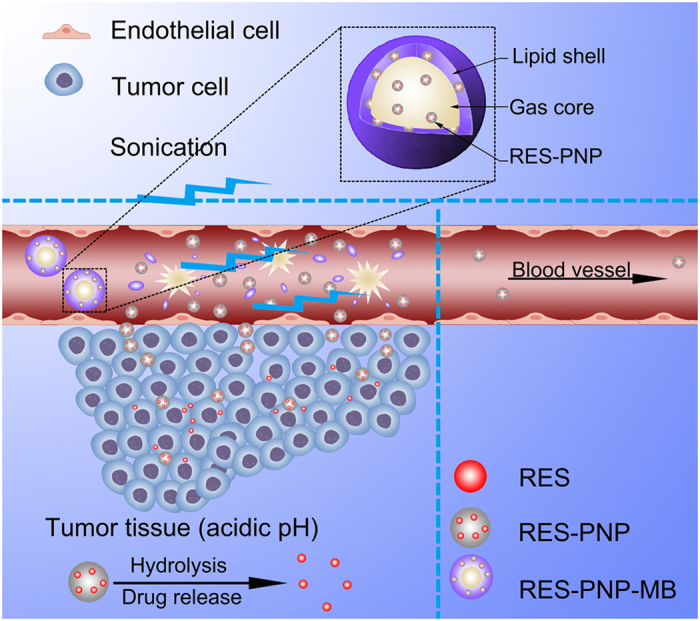
Schematic of RES-PNP-MB as a drug delivery vehicle for the enhanced tumor targeting.

**Figure 2:**
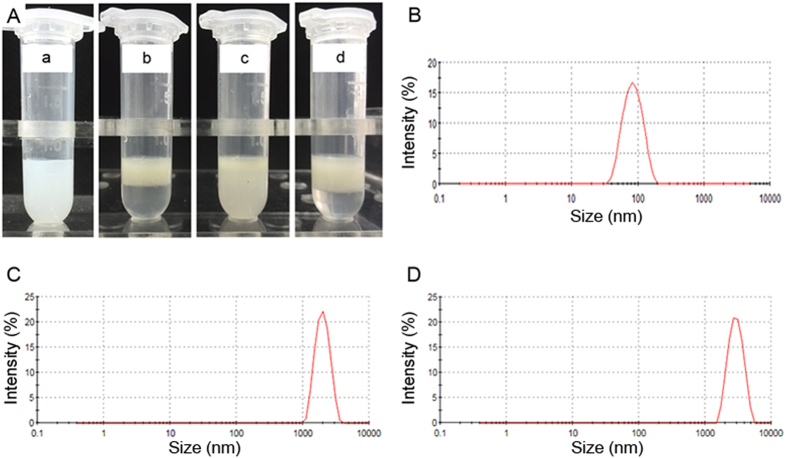
(**A**) PNP-MB after 3 h of standing: (a) PNP; (b) blank microbubbles; (c) physical mixture of microbubbles and PNP; (d) PNP-MB. Size distributions of PNP (**B**), blank microbubbles (**C**) and PNP-MB (**D**).

**Figure 3:**
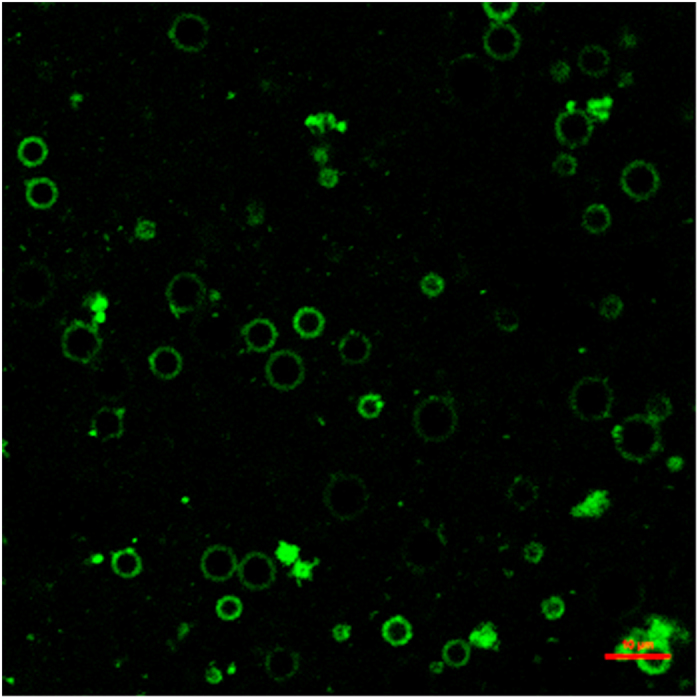
Framework and morphology of PNP-MB observed via CLSM (the scale bar is 10 μm).

**Figure 4:**
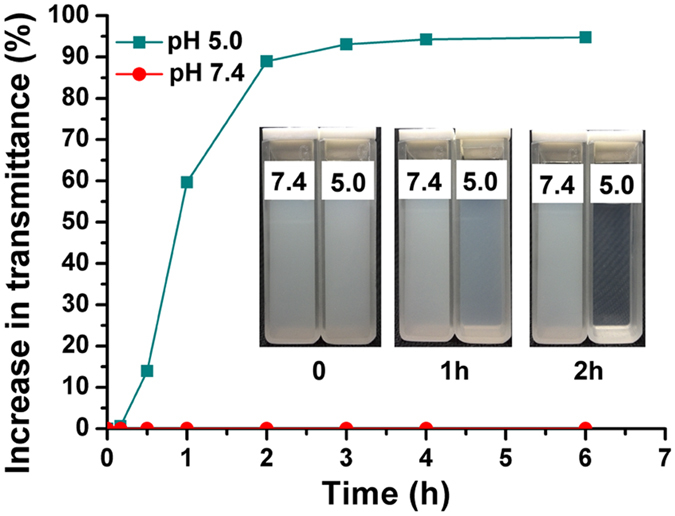
pH-sensitive hydrolysis of PNP in PBS solutions at various pH.

**Figure 5:**
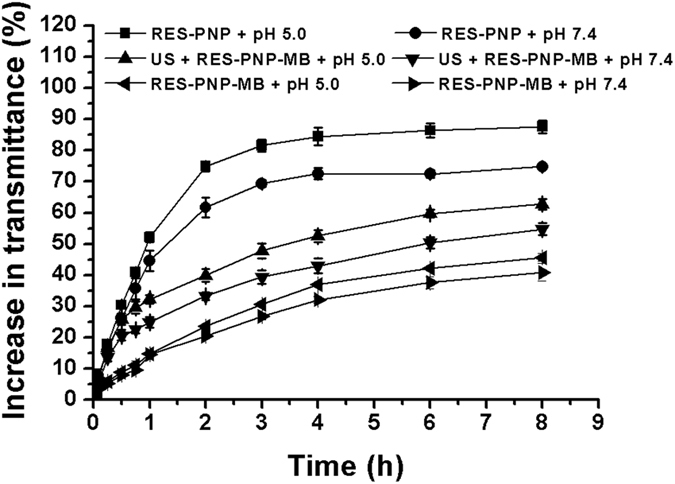
*In vitro* RES release from RES-PNP and RES-PNP-MB in PBS solutions with different pH (*n* = 3).

**Figure 6:**
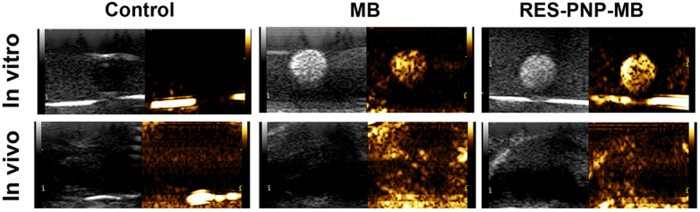
*In vitro* and *in vivo* ultrasound images of degassed and deionized water (control), microbubbles, and RES-PNP-MB.

**Figure 7:**
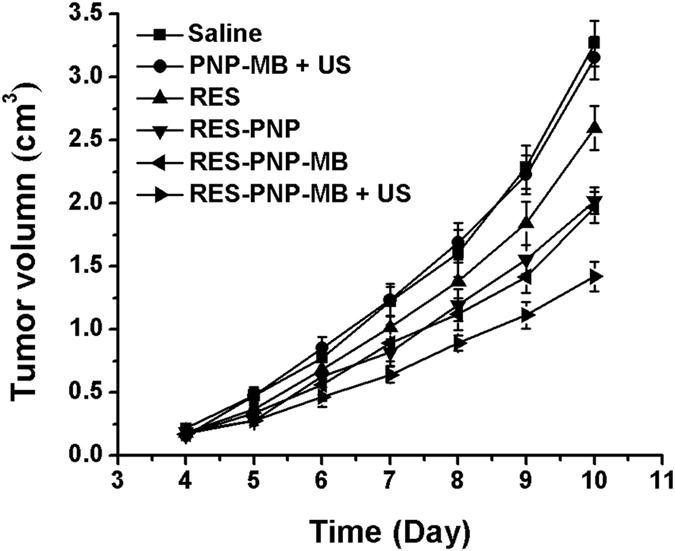
Tumor growth in H22 tumor-bearing mice at 25 mg/kg dosage of RES (*n* = 5). *Note:* The tumor volume of RES-PNP-MB + US compared with those of other groups (*P* < 0.01).

**Figure 8:**
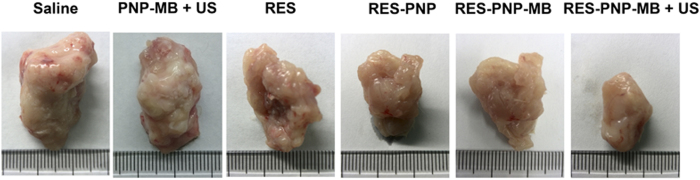
Photographs of H22 tumors from the tumor-bearing mice treated with saline, PNP-MB + US, RES, RES-PNP, RES-PNP-MB, and RES-PNP-MB + US.

**Figure 9:**
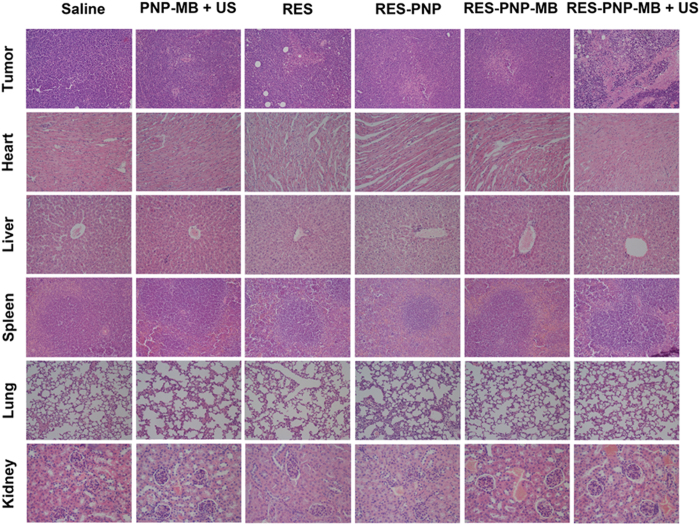
Representative histological (HE staining) images of the tumors and major organs collected on day 10 (original magnification 200×).

The highest tumor inhibition rate, slowest tumor growth and good biosecurity of group VI may be mainly attributed to the combined influence of the EPR effect, the pH sensitivity of PNP, and the tumor site-specific delivery of nanoparticles with the help of ultrasound-targeted microbubble destruction. After RES-PNP-MB was administered (Fig. 1), the lipid membrane of RES-PNP-MB reduced the release and diffusion of drugs from microbubbles during blood circulation before they reached the tumor site. When the RES-PNP-MB reached the tumor site, these vehicles could be detected and destroyed through ultrasound irradiation to release the loaded RES-PNP. RES-PNP entered the tumor blood vessels when the endothelial gaps in the tumor vessel were increased by the combined effect of EPR and microbubble collapse. The degradation of nanoparticles was triggered by the low pH in the tumor interstitium, and the entrapped drug was released. These processes will reduce the systemic toxicity and efficiently enhance the targeting effect of the antitumor drugs (Fig. 1).

## Methods and Materials

### Materials

Soybean lecithin was purchased from Shanghai Advanced Vehicle Technology Co., Ltd. (Shanghai, China). Propanetriol, β-Cyclodextrin (β-CD), and triethylamine (TEA) were obtained from Chongqing Chuandong Chemical Co., Ltd. (Chongqing, China). Pyridinium 4-toluenesulfonate (PPTS), coumarin-6 and rhodamine B were supplied by Aladdin Industrial Inc. (Shanghai, China). Phosphate buffered saline (PBS) and 2-methoxypropene were bought from Sinopharm Chemical Reagent Co., Ltd. (Peking, China). All other chemicals were analytical grade and used as received.

Mouse hepato-carcinoma (H22) cells were kindly donated by Chongqing Key Laboratory of Biochemistry and Molecular Pharmacology (Chongqing, China) and cultured in 1640 medium (Thermo Fisher Scientific Inc., USA) supplemented with 10% fetal bovine serum (FBS).

Male Kunming mice (20 ± 2 g) were obtained from the Laboratory Animal Center, Chongqing Medical University (Chongqing, China) and kept under standard environmental conditions. All experimental protocols were approved by the Institutional Animal Care and Use Committee of Chongqing Medical University. All the experimental operations of animals were carried out in accordance with the protocol approved by the Institutional Animal Care and Use Committee of Chongqing Medical University.

### Synthesis of pH-sensitive material

The acetylated β-CD (A-CD) was synthesized according to a previously reported method^36^. In brief, 80 mg of PPTS was added to 100 mL of anhydrous dimethylsulfoxide (DMSO), and 5 g of β-CD was dissolved under magnetic stirring for 3 min. Subsequently, 20 mL of 2-methoxypropene was added to the solution, and the resulting reaction mixture was stirred for another 3 h at 30 °C before the reaction was terminated by the addition of 2 mL of TEA. The mixture was poured into 400 mL of deionized water to precipitate A-CD. The white precipitate was washed with deionized water until neutral pH was obtained. The product was collected through centrifugation at 12 000 × *g* for 20 min in a centrifuge (Thermo Fisher Scientific Inc., USA) and then freeze-dried.

### Preparation of PNP

The nanoparticles were prepared via an emulsion/solvent evaporation method. In brief, 60 mg of A-CD was completely dissolved in 2 mL of absolute ethanol; afterward, 1 mL of the solution was withdrawn with a 1 mL syringe and added dropwise with vigorous stirring (1000 rpm) to 5 mL of deionized water containing 20 mg of F68 as a surfactant at 30 °C. The emulsion was stirred for 10 min and then transferred into a water bath at 50 °C to allow the ethanol to evaporate with mild stirring (400 rpm) for another 3 h. The nanoparticle suspension was filtered through a 0.45 μm microporous membrane while the suspension cooled to room temperature; the filtrate was freeze-dried to obtain the white powdered nanoparticles (blank PNP).

RES-PNP was prepared using the same methods as those used for blank PNP except 8 mg of RES and 60 mg of A-CD were dissolved in 2 mL of absolute ethanol. The whole preparation process was performed in a dark place.

### Preparation of PNP-MB

The microbubbles and PNP-MB were prepared in accordance with a previously reported method^38^. In brief, 10 mg of lecithin, 100 mg of propanetriol, and 210 μL of PBS or the nanoparticle suspension were sequentially added to a 2 mL EP tube. The mixture was incubated in thermostatic water bath oscillators at 55 °C and 100 rpm for 2 h. The tube was filled with perfluorocarbon gas and mechanically shaken for 45 s on a vortex mixer (Shenzhen baoyu technology Co., Ltd., China) to obtain the blank microbubbles and PNP-MB.

The prepared PNP-MB was washed thrice at 1000 rpm for 3 min to remove the unencapsulated nanoparticles. The floating upper layer was collected and stored at 4 °C for further analysis.

### Characterization of preparations

PNP was labeled with coumarin-6 (C-6-PNP) via the same methods as the preparation of blank PNP except coumarin-6 was dissolved in propanetriol (2 × 10^−6^ M) in advance^39^. The coumarin-6-labeled PNP-MB was then observed through the confocal laser scanning microscopy (CLSM, Nikon A1R, Japan) to investigate the morphological characteristics of PNP-MB.

The diameter distribution and zeta potential of the preparations in deionized water were determined through dynamic light scattering (DLS) in a Malvern Zetasizer Nano ZS instrument (Malvern Instruments Ltd, UK).

### Encapsulation efficiency (EE) of RES-NP-MB

The amount of RES was calculated by dissolving the preparations in absolute ethanol, and the concentration of RES was detected at a wavelength of 306 nm by using a UV-Vis spectrophotometer (UV-5200PC, Shanghai Metash instruments Co., Ltd., China). EE was calculated with the following equations^40^:


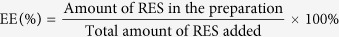


### *In vitro* release and hydrolysis

A dynamic dialysis method was applied to analyze the release of RES from different preparations. In brief, 1 mL of different preparations containing 650 μg RES was sealed in a dialysis bag (MWCO = 8,000–14,000 Da; Spectrum, USA), immersed in 25 mL of PBS (0.02 M; pH = 5.0, 7.4) containing 0.5% (w/w) SDS, and incubated in a thermostatic water bath oscillator (Shanghai sumsung laboratory instrument Co., Ltd., China) at 37 °C and 100 rpm in the dark. At set intervals, 2 mL of the sample was collected to analyze the released RES; the withdrawn volume was replaced with 2 mL of fresh PBS. The released RES was quantified through UV-Vis spectrophotometry, as described in Section 2.4.

PNP was hydrolyzed in two matched cuvettes. In brief, 2 mL of the nanoparticle suspensions was added to each of the two cuvettes. Afterward, 1 mL of PBS at different pH (0.02 M; pH = 5.0, 7.4) was separately added to each cuvette. The transmittance at 500 nm was obtained at a predetermined time point to evaluate the degree of nanoparticle hydrolysis indirectly^36^.

### Animal model

H22 cells were reanimated and cultured in 1640 medium (Thermo Fisher Scientific Inc., USA) for three rounds of subcultivation. Approximately 2 × 10^6^ H22 cells in 0.2 mL of sterilized PBS were injected into the abdominal cavity of male Kunming mice (20 ± 2 g) for serial subcultivation. Ascites were withdrawn from the mice with viable H22 ascite tumor cells and diluted in sterilized PBS to modulate the cell density at 2 × 10^7^ cells/mL. Afterward, 0.2 mL of the diluted H22 cell suspension was subcutaneously inoculated into the right axillary region of each mouse. At 24 h after implantation, the tumor-bearing mice were randomly assigned to several treatment groups, with 5 males in each group, for antitumor analysis.

### Ultrasound imaging

At 7 days after tumor inoculation, the ultrasound images *in vivo* were screened by using a Doppler ultrasonic diagnostic apparatus (Esaote Healthy Co., Ltd, Italy) before and after the mice were injected with 0.2 mL of different formulations. The *in vitro* ultrasound imaging of RES-PNP-MB was performed in 4 mL EP tube. The pictures and videos were captured and recorded for further comparison.

### *In vivo* antitumor analysis

The antitumor analysis was facilitated after 24 h of tumor inoculation. The tumor-bearing mice were randomized into five treatment groups (groups I-VI), with 5 male mice in each group: group I (saline, control), group II (PNP-MB + US), group III (RES), group IV (RES-PNP), group V (RES-PNP-MB) group VI (RES-PNP-MB with ultrasound treatment, RES-PNP-MB + US). Each mouse was injected with 0.2 mL of various formulations with 25 mg/kg/d RES into the tail vein for 9 days.

Ultrasound exposure was performed with an ultrasound transducer (Model UTG 1025, Institute of Ultrasound Imaging of Chongqing Medical Sciences, China) at an intensity of 2 W/cm^2^ for 10 s, with 10 s pauses for a total of 6 min^41^. After 10 days of tumor inoculation, the tumor-bearing mice were sacrificed through cervical dislocation. The tumors were collected, weighed, and immersed in 4% formaldehyde solution to prepare for pathological sectioning with hematoxylin–eosin (HE) staining. The tumor volume was calculated with the following equation^42^: volume = (L × S^2^)/2, where L and S denote the longest and smallest diameters of the tumor, respectively.

### Statistical analysis

The experiments were performed in triplicate. Data were reported as mean ± standard deviations (S.D.) and analyzed with SPSS 13, the probabilities of *p* < 0.05 were considered significant.

## Conclusion

This study fabricated pH-sensitive nanoparticles loaded into microbubbles with the combined advantages of EPR effect, pH responsiveness, targeted treatment, and ultrasound tumor imaging. PNP with controllable size and size distribution was prepared via an emulsion/solvent evaporation method. As an antitumor drug, RES was loaded into PNP to produce RES-PNP that exhibited a pH-sensitive *in vitro* release. Afterward, RES-PNP was successfully loaded into the microbubbles to form RES-PNP-MB that exhibited the combined advantages of nanoparticles and microbubbles. RES-PNP-MB significantly enhanced the antitumor efficacy of RES on H22 tumor-bearing mice. Thus, PNP-MB may be a novel vehicle that can be used for targeted tumor therapy.

## Additional Information

**How to cite this article**: Lv, Y. *et al*. Novel multifunctional pH-sensitive nanoparticles loaded into microbubbles as drug delivery vehicles for enhanced tumor targeting. *Sci. Rep*. **6**, 29321; doi: 10.1038/srep29321 (2016).

## Figures and Tables

**Table 1:** Tumor weight and tumor inhibition rate of H22 tumor-bearing mice exposed to different treatments (*n* = 5).

Group	Tumor weight (mg)	Tumor inhibition rate (%)
Saline	2892.5 ± 345.3	—
PNP-MB + US	2779.7 ± 252.4	3.9
RES	2239.3 ± 232.4^*^	22.6
RES-PNP	1887.2 ± 217.9^*^	34.8
RES-PNP-MB	1875.9 ± 212.6^*^	35.1
RES-PNP-MB + US	1394.7 ± 263.1^*,**,#,*#^	51.8

*Notes*: *p < 0.01 compared with the saline; ^**^p < 0.01 compared with the RES; ^#^*p* < 0.01 compared with the RES-PNP; ^*#^*p* < 0.01 compared with the RES-PNP-MB.
